# Thermocline Regulated Seasonal Evolution of Surface Chlorophyll in the Gulf of Aden

**DOI:** 10.1371/journal.pone.0119951

**Published:** 2015-03-19

**Authors:** Fengchao Yao, Ibrahim Hoteit

**Affiliations:** Division of Physical Sciences and Engineering, King Abdullah University of Science and Technology, Thuwal, Makkah, Saudi Arabia; CNRS, FRANCE

## Abstract

The Gulf of Aden, although subject to seasonally reversing monsoonal winds, has been previously reported as an oligotrophic basin during summer, with elevated chlorophyll concentrations only occurring during winter due to convective mixing. However, the Sea-Viewing Wide Field-of-View Sensor (SeaWiFS) ocean color data reveal that the Gulf of Aden also exhibits a prominent summer chlorophyll bloom and sustains elevated chlorophyll concentrations throughout the fall, and is a biophysical province distinct from the adjacent Arabian Sea. Climatological hydrographic data suggest that the thermocline, hence the nutricline, in the entire gulf is markedly shoaled by the southwest monsoon during summer and fall. Under this condition, cyclonic eddies in the gulf can effectively pump deep nutrients to the surface layer and lead to the chlorophyll bloom in late summer, and, after the transition to the northeast monsoon in fall, coastal upwelling driven by the northeasterly winds produces a pronounced increase in surface chlorophyll concentrations along the Somali coast.

## Introduction

**Fig 1:**
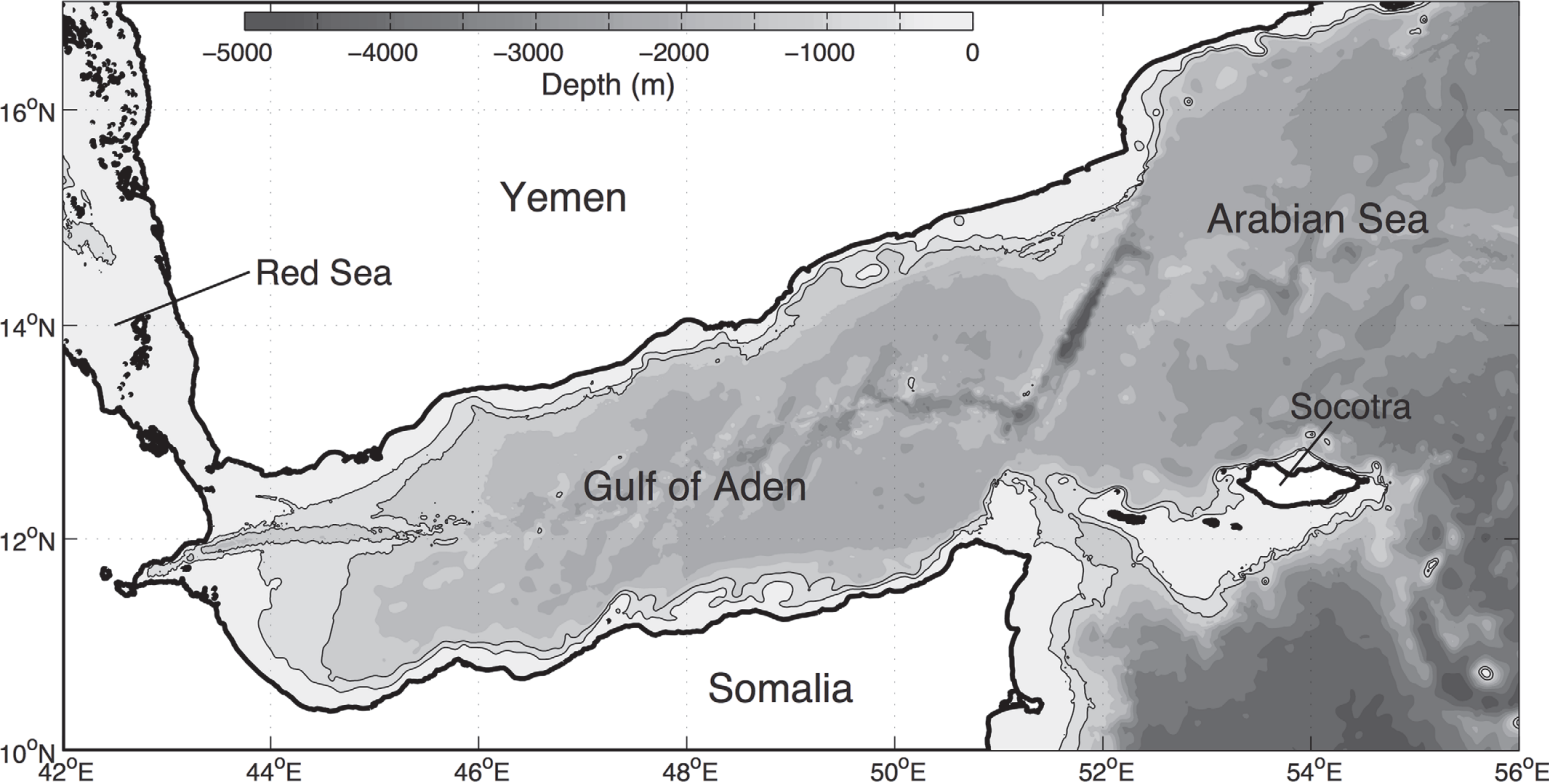
Bathymetry in the Gulf of Aden. The 500- and 1000-m isobaths are plotted on the map in addition to the depth shadings.

The Gulf of Aden is a deep-water basin located between Yemen and Somalia, with a narrow connection to the Red Sea through the Straits of Bab el Mandeb in the northwest and a wide opening to the Arabian Sea in the east (Fig. 1).

Unlike the neighboring Arabian Sea, which experiences intensive summer and winter chlorophyll blooms in response to, respectively, the southwest and northeast monsoons and has attracted intensive studies [1, 2], the Gulf of Aden has been considered, in very limited studies [3, 4], as an oligotrophic area during summer, with a surface chlorophyll bloom only occurring during winter due to deep convective mixing. During summer, the surface nutrient concentrations based on sparse measurements in the Gulf of Aden were reported to be at a very low level, showing practically zero values [4], and coastal upwelling, which is the main physical mechanism inducing the chlorophyll blooms off the Somali and Omani coasts in the Arabian Sea, is not evident in the Gulf of Aden as indicated by the relatively warm surface sea temperature (e.g., see Fig. 2 in [5]).

Nevertheless, relatively high resolution seasonal surveys [6] showed that large-scale upwelling in the Gulf of Aden takes place during the southwest monsoon, resulting in a remarkable ~100-m shoaling of the main thermocline in the entire gulf. As shown in a global study [7], the thermocline in the tropics often coincides with the nutricline, which separates the deep nutrient-rich water from the nutrient-depleted surface water. One important remaining question is how such shoaling of the thermocline in the gulf affects the upward fluxes of deep nutrients and the primary production.

Meanwhile, modeling studies suggested that the basin-scale upwelling, instead of coastal upwelling, is responsible for the cessation of the winter Red Sea outflow and the subsurface intrusion of the Gulf of Aden intermediate water into the Red Sea [8, 9]. Therefore, the Gulf of Aden could be an important nutrient source to the southern Red Sea during the subsurface summer intrusion and a better understanding of the seasonal variability of the vertical nutrient distributions in the Gulf of Aden is also important for the study of the ecosystem in the southern Red Sea [10].

In this study, we use satellite ocean color data to show that, in addition to the winter increase of chlorophyll, the Gulf of Aden exhibits a prominent summer chlorophyll bloom and sustains elevated chlorophyll concentrations during fall. The summer and fall surface chlorophyll evolutions are found indeed to be critically modulated by the seasonal fluctuation of the thermocline in the Gulf of Aden, as revealed by monthly climatological hydrographic and dissolved inorganic nutrient data. In the following sections, the spatiotemporal variability of the thermocline and surface chlorophyll distributions in the Gulf of Aden is described in detail, and the altimetric sea level anomaly (SLA) data and surface wind stress data derived from satellite scatterometer are further used to investigate additional physical mechanisms that help transport the nutrients to the surface and induce the chlorophyll increases.

## Data

The monthly chlorophyll concentration data for 1997–2010 used in this study are derived from the 9-km Sea-Viewing Wide Field-of-View Sensor (SeaWiFS) chlorophyll-*a* ocean color data (http://oceancolor.gsfc.nasa.gov/). The in situ monthly climatological temperature and nutrient data are obtained from the one-degree objectively analyzed World Ocean Atlas 2009 (WOA09) [11, 12]. The monthly SLA data for 1992–2010 with a resolution of one-third degree are derived from the altimetric products that were produced by Ssalto/Duacs and distributed by AVISO. The monthly climatological surface wind stress data are from the one-quarter degree Scatterometer Climatology of Ocean Winds (SCOW) [13], which is based on the 1999–2009 QuikSCAT scatterometer data.

The satellite retrieval of ocean color data in the Gulf of Aden is highly affected by the heavy aerosol loads in this region [14] and so far there are no in situ observations available to assess the quality of the satellite ocean color data in the gulf. A recent study [15] reported a comparison of chlorophyll for the Red Sea between the satellite ocean color data and three in situ measurements taken in October 2008, March 2010 and September-October 2011. The comparison suggests that the accuracy of the standard chlorophyll algorithm for the Red Sea is comparable with that in the global ocean. As the atmospheric aerosol conditions are similar between the Red Sea and the Gulf of Aden, it is reasonable to assume the satellite chlorophyll data for the Gulf of Aden are as accurate as in the Red Sea and other regions in the global ocean.

## Results

### 1. Seasonal Thermocline Structures in the Gulf of Aden

**Fig 2:**
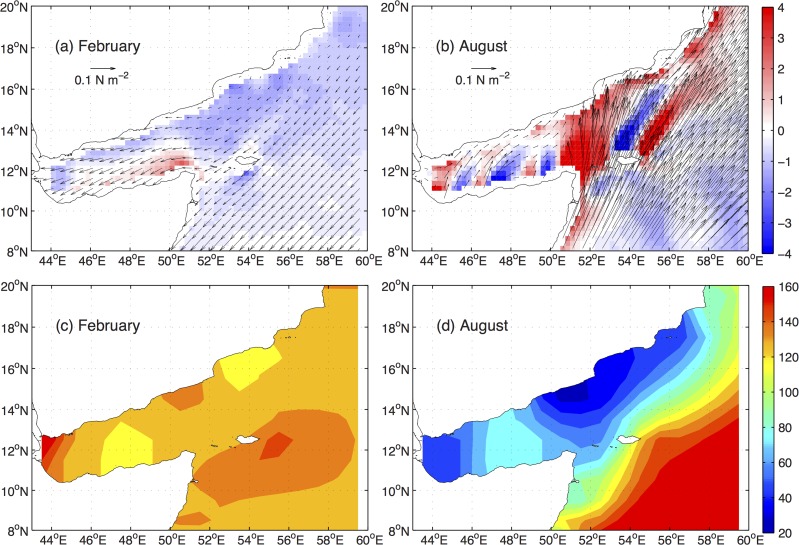
Monthly climatological SCOW wind stress and curl (×10^−7^ N m^−3^) in the Gulf of Aden and western Arabian Sea for (a) February and (b) August, and the WOA thermocline depths (m), represented by the 20°C isotherm, for (c) February and (d) August.

The surface winds in the Gulf of Aden are influenced by the Indian monsoon system and reverse seasonally from northeasterly during the northeast monsoon (from November to April) to southwesterly during the southwest monsoon (from June to September). The surface winds in the gulf during the northeast monsoon are similar to those in the Arabian Sea (Fig. 2A). During summer, the Gulf of Aden experiences much weaker surface winds than the Arabian Sea because it is located off the axis of the strongest southwest monsoon, the Findlater Jet, and is shielded from the mountains along the Somali coast, which also produce an alternatingly positive and negative pattern in the wind stress curls due to orographic effect.

It is well established that the thermocline in a broad region off the coast of the Arabian Peninsula in the Arabian Sea is drastically lifted upward during the southwest monsoon from the winter condition, due to the upward Ekman pumping associated with the positive wind stress curls at the left side of the Findlater Jet axis [16]. The response of the thermocline in the Arabian Sea to the southwest monsoon is well reflected in the climatological SCOW wind stress data and the WOA09 hydrographic data shown in Fig. 2, with shoaling thermocline depths (represented by the 20°C isotherm) corresponding to the positive wind stress curls along the Arabian coast and deepening thermocline depths corresponding to the negative wind stress curls in the central Arabian Sea. The small-scale negative wind stress curls at the lee of Socotra Island appear to have a limited effect on the large-scale thermocline shoaling, but are considered to be the formation mechanism for the anti-cyclonic Lee Eddy there observed in the AVISO SLA data [6].

However, the shoaling of the thermocline is not limited to the region off the coast in the Arabian Sea, but also in the entire Gulf of Aden with a similar magnitude, about 100 m, (Fig. 2D), which is consistent with the basin-wide surveys in [6]. Because the wind stress curls in the Gulf of Aden are relatively weak with a variable pattern, the shoaling of the thermocline in the Gulf of Aden does not appear to be a response to the local surface winds. Instead, it was attributed to the eastward Ekman transport driven by the southerly winds at the eastern opening of the gulf in a numerical study [8]. Surface drifter data also revealed a surface outflow from the Gulf of Aden during the southwest monsoon [15], implying that a balancing deep outflow from the Arabian Sea exists and the shoaling of the thermocline is driven by a baroclinic water exchange between the Gulf of Aden and the Arabian Sea.

**Fig 3:**
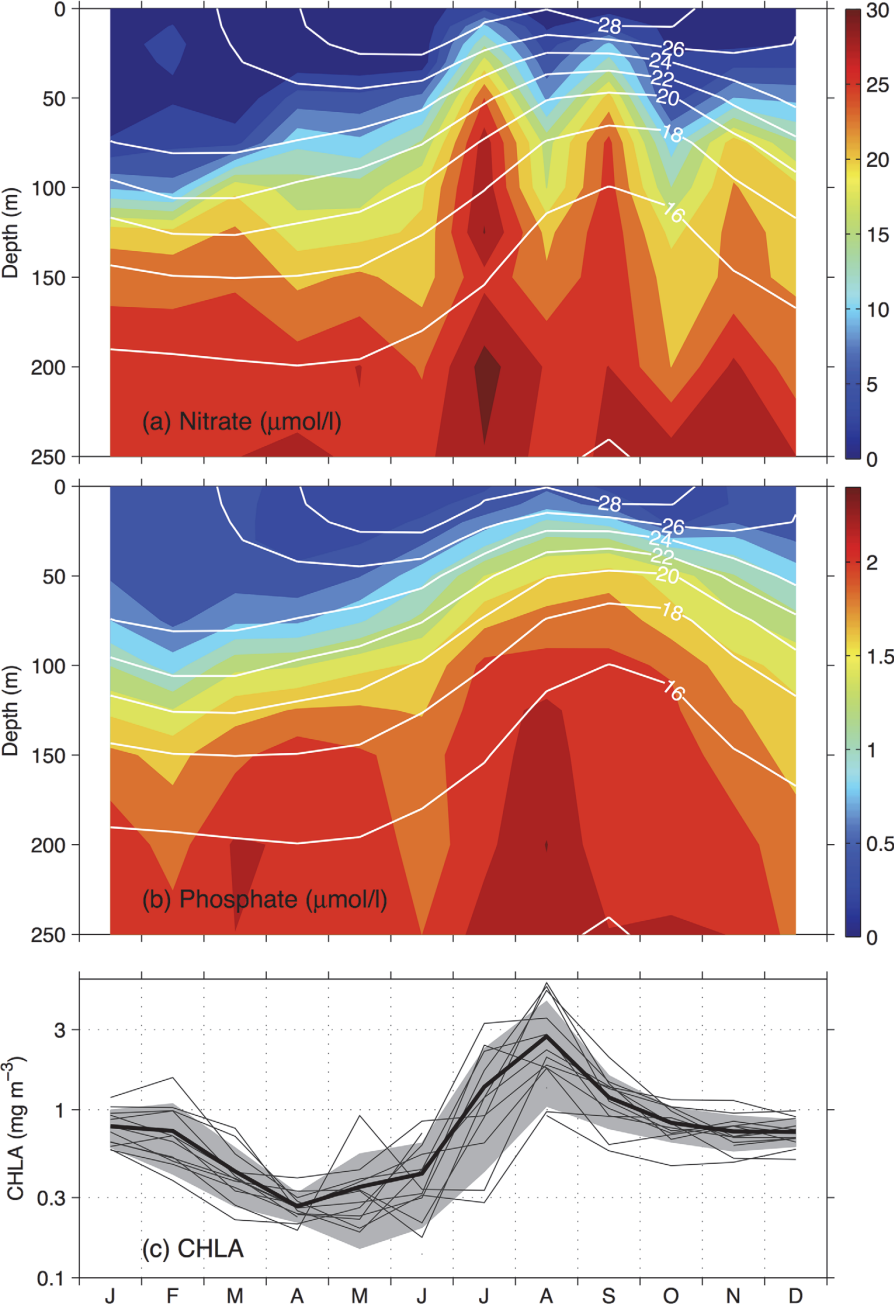
Seasonal cycles of the WOA vertical profiles averaged in the Gulf of Aden for (a) nitrate and (b) phosphate, with temperature contours (°C) superimposed, and (c) the SeaWiFS chlorophyll-*a* (CHLA) concentrations averaged in the Gulf of Aden for 1998–2010 shown in logarithmic scale, with the thin lines denoting individual year values, the thick line denoting the climatologically mean values, and the shaded envelop denoting the ±1 standard deviations.

It was suggested that the open-ocean upwelling associated with the Ekman pumping is a critical physical process for the elevated biological productivity during August-September in the Arabian Sea [16]. Here, the effects of the large-scale upwelling and the resulting shoaling of the thermocline in the Gulf of Aden are examined in Fig. 3, where the seasonal cycles of the WOA09 vertical profiles of temperature, dissolved inorganic nutrients, including nitrate and phosphate, averaged over the Gulf of Aden are shown.

From the onset of the southwest monsoon in May, the upwelling in the Gulf of Aden induces an upward displacement of cold, deep water as indicated by the rising isotherms (Fig. 3). The thermocline (represented by the 20°C isotherm) reaches the shallowest depths during August-September and stays uplifted until December. The upward movement of the thermocline has a significant effect on the vertical nutrient distributions. During the winter condition from January to May, the nitrate and phosphate are depleted in the upper euphotic zone and the vertical distributions exhibit a sharp gradient at the base of the euphotic zone, the depth of which is about 100 m during this period according to estimates from the SeaWiFS data. Following the upward movement of the thermocline, significant upward nutrient fluxes occur in the gulf, replenishing the euphotic zone with deep nutrients. Despite that the deep nitrate reaches its shallowest depths in July and September and the phosphate in August-September, it is noteworthy that the nutrients in the upper layer are still at higher levels from October to December after the transition to the northeast monsoon when compared with the winter condition.

### 2. Seasonal Distributions of the Surface Chlorophyll in the Gulf of Aden

The seasonal cycles of the SeaWiFS surface chlorophyll concentrations in the Gulf of Aden averaged for the period from 1998 to 2010 are displayed in Fig. 3C. Despite the marked interannual variability, especially during summer when uncertainty is high due to poor data coverage caused by heavy clouds, a consistent mean seasonal pattern in the surface chlorophyll concentrations emerges.

After the low-productivity spring (from late March to early June), the SeaWiFS data reveal a prominent chlorophyll summer bloom (from July to September) and elevated chlorophyll concentrations throughout the fall (from October to December), in addition to the winter (from January to early March) increase in the chlorophyll concentrations. It can be seen that the evolution of summer and fall surface chlorophyll concentrations are closely linked to the seasonal cycles of the thermocline and the vertical nutrient distributions (Fig. 3A and 3B). The summer bloom develops rapidly from June when the thermocline has started shoaling, and reaches the peak value of 2.7 mg m^−3^ during August when the thermocline is at the shallowest depth. The summer bloom is followed by a plateau of chlorophyll concentrations of ~0.8 mg m^−3^ from October to December when the thermocline is still at shallower depths than during winter.

At the same time, further examinations of the relation between the surface chlorophyll concentrations and the vertical profiles of nutrient suggest that the biological processes also play an important role in controlling the temporal variability of the surface chlorophyll concentrations. For instance, the decrease in nitrate in the upper layer during August (Fig. 3A) is probably due to a large increase in consumption by blooming phytoplanktons, and the quick decline of the surface chlorophyll concentrations in September, when both nutrients are still at very high concentrations in the upper layer, is probably due to top-down control by zooplanktons.

The seasonal cycle of the surface chlorophyll in the Gulf of Aden is similar to that in the northwestern Arabian Sea shown in [2], both in the timing of development and the peak chlorophyll concentrations, but, as discussed below, the driving physical mechanisms for the summer and fall surface chlorophyll evolution in the gulf are not the same as in the northwestern Arabian sea, where coastal and open-ocean upwelling are the main driving mechanisms [1, 2].

**Fig 4:**
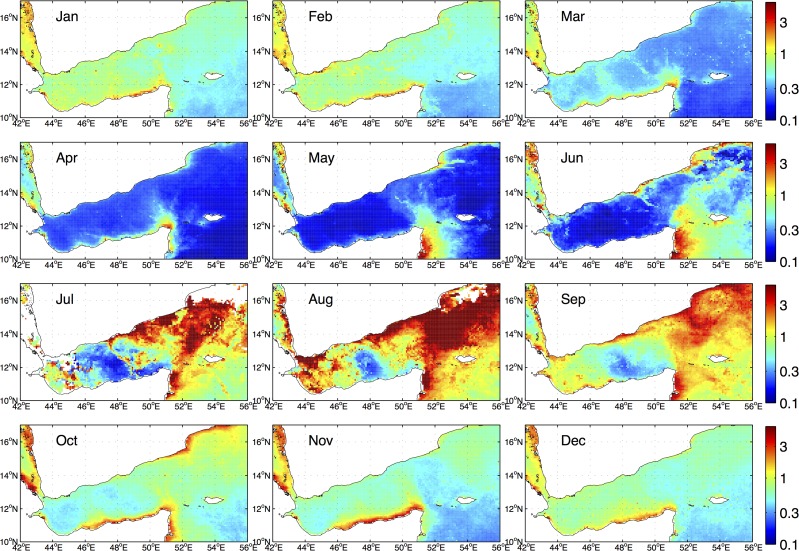
Seasonal distributions of the climatological SeaWiFS chlorophyll concentrations (mg m^−^^3^) in the Gulf of Aden and the western Arabian Sea.

The climatological monthly surface chlorophyll distributions are shown in Fig. 4 to depict the seasonal evolution of the surface chlorophyll in the Gulf of Aden. Four distinct spatial patterns from the 12 monthly plots can be identified: winter (from January to March), spring (from April to June), summer (from July to September) and fall (from October to December), with each pattern reflecting substantially different underlying physical processes.

The winter (from January to March) surface chlorophyll shown in Fig. 4 is characterized by scattered patches of increased chlorophyll levels; a weak stratification in temperature in the upper 100 m during January and February shown in Fig. 3A and 3B suggests that the dominant driving mechanism during the winter increase of chlorophyll concentrations is the upward nutrient fluxes by entrainment associated with the deep convection. The surface chlorophylls in spring (from April to June) show very low concentrations (< 0.3 mg m^−3^) in the whole gulf. Even though no subsurface data are available for the whole euphotic zone, the spring condition for the gulf is likely to be oligotrophic, as there are no evident physical processes to supply nutrients to the surface layer because the water column becomes more stratified and the locations of the thermocline and nutrientcline remain at deep depths.

The summer (from July to September) distributions exhibit a persistent pattern with surface chlorophyll blooms appearing in both the western and the eastern parts of the basin and a contrasting oligotrophic region in the central part around 48°E. This pattern is mainly related to the mesoscale eddies as discussed later. During fall (from October to December), elevated chlorophyll levels are mainly found along the Somali coast, and the different spatial pattern in the fall distribution as compared with the summer bloom indicates that the former is not a continuation of the latter. Instead, the fall chlorophyll distributions in the Gulf of Aden are consistent with the coastal upwelling driven by the northeasterly winds during the northeast monsoon (see Fig. 2A for the February surface winds, which remain basically the same during the northeast monsoon). Furthermore, the marked contrast of the surface chlorophyll between fall and spring highlights the importance of the upward nutrient fluxes associated with the shoaling of the thermocline after the reversal of the southwest monsoon, given that the gulf is subject to similar northeasterly winds during both fall and spring.

As no significant coastal upwelling is observed during the summer bloom and the advection of surface chlorophyll from the northwestern Arabian Sea is unlikely due to the presence of a surface outflow from the gulf during summer [17], the effects of meso-scale eddies on the upward nutrient fluxes are further examined as an additional physical mechanism supplementary to the thermocline shoaling in the gulf.

Mesoscale eddies are observed from the SLA and drifter data in the Gulf of Aden throughout the year [6]. These eddies are either generated outside the Gulf of Aden and propagate westward into the basin during the northeast monsoon [18, 19], or generated locally during the southwest monsoon by Ekman pumping associated with positive and negative wind stress curls (Fig. 2B) in the Gulf of Aden and remain mostly stationary [6]. The effects of mesoscale eddies on the subsurface density surfaces, with the cyclonic eddies lifting up the density surfaces and the anti-cyclonic eddies depressing them, are an important mechanism of upward transport of deep nutrients [20]. It was also showed there is a strong spatial correlation between the mesoscale eddies and the chlorophyll distributions in the Gulf of Aden [18].

**Fig 5:**
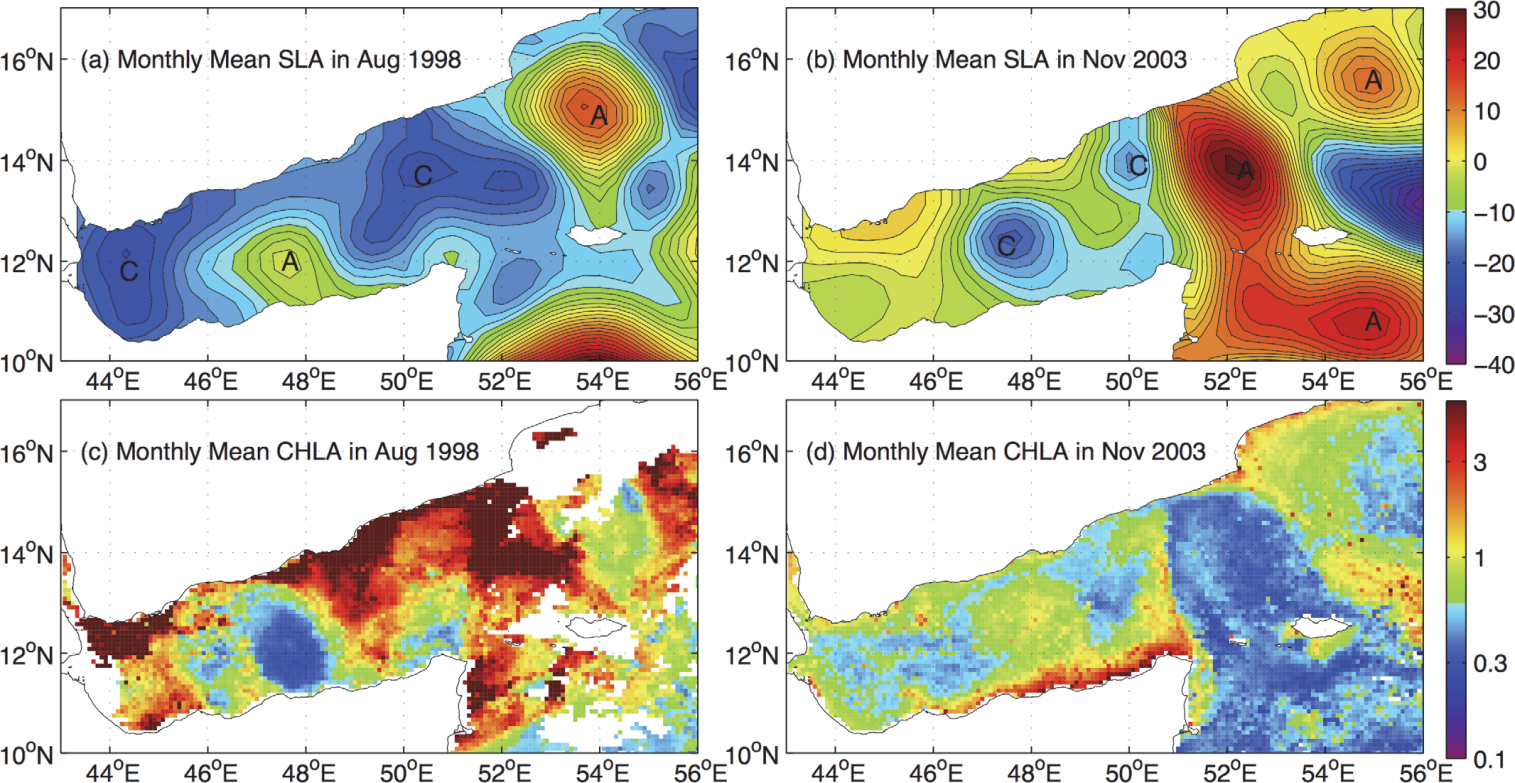
Impact of mesoscale eddies on the distributions of surface chlorophyll-*a*: monthly mean sea level anomalies (SLA, cm) in (a) August 1998 and (b) November 2003, and monthly mean chlorophyll-*a* (CHLA) concentration (mg m^-3^) in (c) August 1998 and (d) November 2003. The cyclonic and anti-cyclonic eddies as suggested by the SLA are marked as “C” and “A”, respectively.

The effect of meso-scale eddies on the summer and fall surface chlorophyll concentrations in the Gulf of Aden are examined with a synthesis of the AIVSO SLA data and the SeaWiFS data in Fig. 5. The monthly SLA and chlorophyll data for August 1998 and November 2003 provide relatively complete data coverage and are used to exemplify the effects of the meso-scale eddies. For the monthly data in August 1998 (Fig. 5A and 5C), a strong spatial correlation is evident, with an anti-cyclonic eddy corresponding to the low chlorophyll region in the middle of the basin, and cyclonic eddies corresponding to the elevated chlorophyll levels in the western and eastern parts of the basin. The August 1993 section observed along the axis of the Gulf of Aden (Fig. 4 in [6]) clearly shows the distortions of the isotherms by the eddies, with the cyclonic eddies further lifting up the already shoaled thermocline. The anti-cyclonic eddy around 48°E during August (Fig. 5A), referred to as the “Summer Eddy” in [6], is generated and sustained by the negative wind stress curls (Fig. 2B) in the middle of the gulf and manifests itself annually from July to September in the satellite SLA data shown in [6]. It counter-acts locally the shoaling of the large-scale thermocline in the gulf, resulting in a persistent oligotrophic region in the central gulf as shown in the climatological chlorophyll distribution (Fig. 4). The in situ observations in [4] were measured inside the oligotrophic anti-cyclonic eddy around 48°E, and this explains the discrepancy between the in situ observations indicating a low summer biological productivity and the remote sensing data in this study showing a prominent chlorophyll bloom.

The effects of the cyclonic eddies on the surface chlorophyll is modulated by the mean thermocline depth in the gulf. As the thermocline drops substantially from the shallowest depths and the nutrients in the upper layer are subsequently reduced, the cyclonic eddy around 48°E in November 2003 induces a relatively weak increase of chlorophyll. At the same time, the surface winds along the Somali coast becomes stronger than those along the Yemeni coast during the southwest monsoon (Fig. 2), and the coastal upwelling along the Somali coast forced by the northeasterly appears to be the main driving mechanism for the elevated chlorophyll concentrations along the coast in fall (Fig. 5D).

## Discussion

It is shown in this study that the Gulf of Aden, although subject to similar seasonally reversing monsoonal winds, is a separate biophysical province from the northwestern Arabian Sea, exhibiting distinct spatiotemporal variability in the surface chlorophyll distributions that are controlled by different physical mechanisms. The SeaWiFS ocean color data reveal that, contrary to previous studies, the Gulf of Aden experiences a summer surface chlorophyll bloom and a sustained fall surface chlorophyll concentration increase in addition to the well-known winter bloom.

The climatological hydrographic data indicate that the shoaling of the thermocline, therefore the nutricline, in the entire gulf during summer and fall is of central importance by providing upward nutrient flux and preconditioning the summer and fall surface chlorophyll increases. During summer, as the thermocline and nutricline reach the shallowest depths, facilitated further by vertical nutrient pumping associated with the cyclonic eddies, the surface chlorophyll concentrations reach the peak values in the annual cycle. During fall, when the thermocline and nutricline drops but still remains uplifted and the northeast monsoon prevails in the Gulf of Aden, the surface chlorophyll concentrations increase along the Somali coast as a result of coastal upwelling.

The upward nutrient fluxes caused by the thermocline shoaling have geographically wider implications than the Gulf of Aden. First, the aforementioned summer subsurface intruding Gulf of Aden intermediate water would provide a substantial amount of influx of nutrients to the Red Sea, because the intruding water in the Red Sea has an observed temperature range of 16.5–20.0°C [9, 21], and is apparently supplied by the nutrient-rich water upwelled in the gulf as shown in Fig. 3. Indeed, there was some observational evidence indicating the supply of the nutrient to the southern Red Sea by the subsurface intruding water: The nitrate concentrations observed in early October 1982 showed a fivefold increase in the southern Red Sea [22]. As there is no significant river charge into the Red Sea, the subsurface nutrient supply to the Red Sea is vital for the nutrient budgets in its basin. In this sense, the Gulf of Aden acts as a conduit for the southwest monsoon in the Arabian Sea to remotely influence the biological productivity in the Red Sea.

Second, the sustained increase of surface chlorophyll concentrations during fall in the gulf can be seen as a combined result of the northeast monsoon and the persisting effect of the southwest monsoon on the thermocline structure during fall. As the seasonal variations of thermocline structure in the gulf and in the northwestern Arabian Sea are nearly synchronized (Fig. 2), the results in this study may shed some light on the effects of the persisted shoaling until late fall of the thermocline and nutricline on the surface chlorophyll in the northwestern Arabian Sea, where high-level surface chlorophyll concentrations are sustained for a prolonged period after the withdrawal of the summer coastal upwelling [1, 2].

As the satellite ocean color data and climatological hydrographic data shown in this study reveal the basic physical processes that regulate the seasonal evolution of the surface chlorophyll in the Gulf of Aden, in situ measurements that cover the entire basin with resolutions to resolve the highly variable spatial patterns are required to provide field validations against the remotely sensed results presented in this study. Furthermore, modeling studies that integrate both the physics and biological processes are required to provide more complete insights about the mechanisms that govern the temporal variability of the surface chlorophyll on various spatial and temporal scales.
